# Medical and Physical Intelligence

**Published:** 1805-12

**Authors:** 


### MEDICAL AND PHYSICAL INTELLIGENCE.

### [ FOREIGN AND DOMESTIC. ]

*A new Theory of the Effects of Medicines.*—Dr. RASORI, of Italy, having examined the modern theory, according to which all medical and dietetical substances possess the power of stimulating or inciting the body, found this doctrine too narrowly limited, and that there are certain substances which are debilitating in a very high degree, without previously inciting the body in any particular manner. The Doctor calls these, *contra stimulantia directa.* This assertion he proves by his observations on the effects of the lauro-cerasus. All the animals to whom a large dose of the laurel water was given, became suddenly debilitated, nearly to death. By the application of stimulating medicines they were again restored.—These observations being made upon animals only, were neglected; but some time after, Dr. Borda, an eminent practitioner in Lombardy, prescribed the water of laurocerasus for his patients, and found the same experiments confirmed. Too strong a dose of the water brought on a state of palsy, and the most volatile stimuli were required. Dr. Borda afterwards examined a great number of drugs, the whole stock of which he ranks under two classes, viz. Stimulantia and Contra-stimulantia. To this last class belong, besides the laurocerasus, the bitter almonds, nux vomica, digitalis purpurea, solanum nigrum and dulcamara, gratiola, scilla, antimonialia, the neutral salts, all the acids, and perhaps mercury.

---

From various experiments made in Germany, it appears that the bark of that species of the service tree called by Linnæus *sorbus accuparia*, is well adapted to the tanning of leather, and that six pounds of this bark, collected in autumn, furnishes as much tanning-matter as seven pounds of oak-bark; and ardent spirit may be likewise collected from the ripe berries of this tree. Twelve pounds of berries yield two quarts of spirit; the pulp, after distillation, affords excellent nourishment for cattle.

---

A new thermometer has been invented for registering the highest and lowest temperatures in the absence of the observer, which is said to be a more simple, as well as a less expensive, instrument than Lix's thermometer. It consists in two thermometers, one mercurial, and the other of alcohol, having their stems horizontal.—The former has for its index a piece of magnetical steel wire, and the latter a minute thread of glass, having its two ends formed into small knobs by fusion in the flame of a candle. The magnetical bit of wire lies in the vacant space of the mercurial thermometer, and is is pushed forward by the mercury whenever the temperature rises and pushes that fluid against it; but when the temperature falls, and the fluid retires, this index is left behind, and shews the *maximum*. The other index, or bit of glass, lies in the tube of the spirit-thermometer immersed in the alcohol, and when the spirit retires by the depression of temperature, the index is carried along with it in apparent contact with its interior surface; but on increase of temperature the spirit goes forward and leaves the index behind, which therefore shews the *minimum* of temperature since it was set. The steel index is easily brought to the mercury by applying a magnet on the outside of the tube, and the other is properly placed at the end of the column of alcohol by inclining the whole instrument.

---

As toad stools and other species of the fungus kind are frequently eaten for mushrooms, a method of preventing the pernicious effects has been practised in France, which is stated to be an infallible remedy: — "Excite vomiting, employ laxatives and clysters, and after the first evacuations administer a drachm of sulphuric ether in a glass of water of marsh-mallows. If the symptoms are very alarming, it may be necessary to give a clyster made with a strong decoction of tobacco."

---

The following is the method adopted in Paris of making balsamic and anti-putrid vinegar:—Take the best white-wine vinegar, a handful of lavender, leaves and flowers, the same quantity of sage, leaves and flowers, hyssop, thyme, balm, savory; a good handful of salt, and two heads of garlic; infuse these in the vinegar a fortnight or three weeks; the longer the better; and then it is found to be an excellent remedy for wounds, for spasms and suffocation. By rubbing the hands and temples with it, a person may go into foul air with great safety.

---

A correspondent, in a letter to the Editors, observes, "A writer in the last Medical Review, under the article of Smyth versus Chaptal, makes the following unqualified assertion.—'There is no evidence that any person had ever employed the mineral gases for destroying contagion in the apartments of the sick, prior to the use of them by Dr. Smyth, at Winchester, in the year 1780.' How far this assertion may be correct, I shall leave to be determined by the following quotation from a book published, above sixty years ago, by Gaubius.

"Vapor antiloimicus: ℞ Acet. vin. vulg. lbj: sal. marin; nitri: ol. vitriol. vulg. aa. lbfs: aq. puræ. lbj. M.

"Immissa, in ollam fictilem vitreatam, repandam, super pruinas reposita, in limine domus aut *cubiculi* leniter evaporent.

---

Dr. Davis, of Sheffield, is engaged in a translation of Pinel's Treatise on Insanity, which will be published early in February.

---

Dr.

Dr. Walker, in a letter to the Editors, dated Nov. 23, 1805, says, "I send you the following note, which I received this morning. It will give you pleasure to show that the nobleman, who has all along taken the greatest interest in the discovery of Jenner, still continues his zealous services in the cause of humanity.

"Sir,

"I am very much obliged to you for the vaccine matter, but will not trouble you for any more, as we are now well supplied. I wish the opposers of the cow-pox could see how little their arguments avail, when a death or two by the small-pox occurs. They are rushing from many miles round to be vaccinated, and already some hundreds have been placed in security, so that those two poor victims, above alluded to, have not died in vain. I am,

"Sir, your most obedient humble servant,

*Petworth, Nov.* 21, 1805. "EGREMONT."

---

Dr. Patterson, of Londonderry, is preparing for publication Disquisitions concerning Pestilential and Epidemic Diseases, with a view to obtain valid Principles whereon to found a civil Constitution of Medical Police for Ireland.

To every one conversant in this field of inquiry, it is manifest, that the climate of the country, the physical and moral attributes of the inhabitants, their political and commercial relations, their economical institutes, and other corresponding circumstances, must be taken into consideration in a work of this description. It will also be necessary to take a retrospective survey of the numerous, malignant, and reigning distempers, which have prevailed in many parts of the earth throughout a series of ages. These are not easy tasks, they are not small undertakings; yet difficult and extensive as they are, little short of their atchievment will answer the purpose; a purpose which has, in contemplation, objects of the first national importance: for the aim of those disquisitions is no less than to provide rational grounds on which the public may think and act, in respect to pestilential and contagious disorders; and to lay before practitioners in physic a methodical view of principles, whereon may be erecsed a concentrated and concordant system of practice.

Thus would be suggested, means for obtaining the most desirable ends; the public mind would be supplied with approved sentiments on the subject, and would uot be distracted by vague and silly notions, nor be so liable as at present to fall a prey to ignorance and prejudice; and the medical faculty would have materials for a general plan, according to which they might be induced to proceed in concert, to the honour of themselves, and the advantage of the community; whilst the constituted authorities would be furnished with grounds for acting with the necessary decision, firmness, and effect. How earnestly this state of things ought to be wished and sought for, will at once be felt, if it be considered what affliction and calamities are experienced in the places that are visited by those unrelenting foes to the human race, race, where the people are unacquainted with their nature, are unprepared for their assault, and professional men are at variance both in doctrine and practice. In all cases of danger, unanimity in opinion is the first step towards preservation.

But it may be asked, would not a publication of this kind be a species of trespass on the board of health, lately instituted in Ireland? No—For, instead of interfering with the objects of that institution, the intended work is calculated to correspond with those objects; a design which it would answer by circulating accumulated information on contagious and spreading distempers, in this wise preparing the public mind for receiving official doctrines, and for obeying the peculiar laws with promptitude and efficacy.

At the same time, let it not be imagined that the writer presumes to impose, upon professional gentlemen, a code either of theory or of practice: he forms no such confident opinion, nor makes he such an arrogant attempt: he merely collects facts and observations, arranges them, compares them, weighs them, and, to the best of his judgment, draws from them legitimate conclusions. Thus does he endeavour to present to the mind a store of information, to go along with the reason, to convince the understanding, and ultimately to harmonize and settle the practice in cases of such general interest. By this process of the intellectual faculties, has he formed his own sentiments on the subject. He is likewise encouraged to make this offering to the intellect of others, by finding himself in possession of *very ample materials*, especially derived from the Western World, where malignant epidemics have of late years reigned with extensive and desolating sway. He is further induced to embark in this undertaking, by reflecting, that it does not accord with the situation and pursuits of many individuals, who practise medicine, to obtain a proportionable knowledge of scattered and important facts, without which their practice must be fluctuating, inadequate—most probably pernicious. This radical want he particularly professes to supply; and he trusts, that his medical brethren will take in good part his labours in their service.

Perhaps, in no object of medical research does there exist so great a necessity, as in that before us, for separating the facts observed from the ideas, which, in order to explain those facts, may occur to the mind of the observer. Exclusive of the prejudice which observers are known too often to take up for their own observations; we should consider, that in the present immature state of knowledge relative to the nature of malignant and contagious distempers, every well ascertained fact increases our incompetent stock of information; whilst, on the contrary, the reasonings we are for the most part enabled to advance, are at best but ingenious conjectures, which too often bias and mislead the judgment. It shall therefore be a principal intent of the work here announced, to give facts unmixed with systematic speculations, and in due course to offer such reasonings as shall seem to be fairly sanctioned by the premises, and by the principles of the several sciences that may may appear to be concerned in the procedure. To this work, where facts and reasonings are thus classed, where the concord of some descriptions of men, and the benefit of all are consulted, may it not be expected that a favourable reception will be given? Without this distribution of materials, and the organized system of harmony here contemplated, if the malignant foe arrive, the melancholy consequence will be a national orchestre of woe-toned instruments, playing the "*Dance of Death.*

*October* 8, 1805.

---

Mr. MILBURNE has engaged to instruct a limited number of pupils on a plan calculated to expedite the attainment of professional knowledge. The structure and physiology of the human body will be explained with the operations of Surgery. Gun Shot Wounds will be more particularly described, and a variety of interesting Cases read, as minuted by different medical officers during campaigns on the continent, East and West Indies, America, engagements by sea, &c. Particulars may be had of Mr. Milburne, No. 30, Conduit-street, Hanover-square.

---

Mr. YOUNG, of North Audley-street, will give a Course of Lectures on the Nature and Treatment of Cancer, early in February 1806; a Prospectus of which will be published in the course of next month.

---

Dr. Adams has published a second edition of his "Answers to all the Objections hitherto made against the Cow-Pox," in which he has adverted to such as have been started since the first edition.

---

Dr. JACKSON will shortly publish the First Part of Practical Observations on the Febrile Diseases in Gibraltar, which prevailed so fatally at that place last autumn.

---

The MEDICAL SOCIETY of LONDON beg leave to inform the Medical Public, that any paper in Answer to the first Question for the Fothergillian Medal for the Year ensuing, will be received at the Society's house, Bolt-court, Fleet-street, any time previous to the 31st day of January, 1806.

M. F. WAGSTAFFE, Register.

---

A Meteorological Reader of the Medical and Phyfical Journal will be very much obliged to Dr. HIGGINS, if he will favour the public with an accurate and concife defcription of the Centegrade and Hygrometer, which he ufes in keeping his Monthly Meteorological Table.

